# Enhanced efficacy of immune checkpoint inhibitors combined locoregional therapy and tyrosine kinase inhibitors in the treatment of unresectable hepatocellular carcinoma: A single - center retrospective study

**DOI:** 10.3389/fonc.2025.1554711

**Published:** 2025-02-25

**Authors:** Junfeng Bu, Zihan Li, Die Hu, Ling Lan, Jiwei Huang, Xin Wang, Qiu Li, Jin Zhou, Yong Zeng

**Affiliations:** ^1^ Department of Liver Surgery, West China Hospital, Sichuan University, Chengdu, China; ^2^ West China School of Medicine, Sichuan University, Chengdu, China; ^3^ Department of Radiation Oncology, West China Hospital, Sichuan University, Chengdu, China; ^4^ Department of Medical Oncology, West China Hospital, Sichuan University, Chengdu, China

**Keywords:** unresectable hepatocellular carcinoma, locoregional therapy, tyrosine kinase inhibitor, immune checkpoint inhibitor, system therapy

## Abstract

**Background:**

Unresectable hepatocellular carcinoma (HCC) presents significant treatment challenges. While locoregional therapies (LT) and tyrosine kinase inhibitors (TKI) offer some benefits, prognosis remains poor. Immune checkpoint inhibitors (ICI) have shown promise in other oncological settings, suggesting potential benefits in HCC treatment regimens.

**Methods:**

This retrospective study analyzed 232 patients diagnosed with unresectable HCC at West China Hospital from January 2019 to December 2023. Patients were categorized into two treatment groups: LT+TKI and LT+TKI+ICI. All patients underwent standardized locoregional treatments and first-line TKIs, with the latter group also receiving ICIs. The primary endpoints measured were overall survival (OS) and progression-free survival (PFS). Survival analysis utilized Kaplan-Meier estimates and Cox regression models.

**Results:**

The LT+TKI+ICI group demonstrated significantly improved survival outcomes compared to the LT+TKI group. Median OS was 28 ± 3.9 months in the LT+TKI+ICI group versus 21 ± 3.0 months in the LT+TKI group, with corresponding 6-, 12-, and 24-month OS rates of 96.8%, 79.3%, and 59.4% versus 85.8%, 71.5%, and 44.1%, respectively (HR, 0.64; 95% CI, 0.449-0.913; P = 0.014). Median PFS also favored the LT+TKI+ICI group (11 ± 1.1 months vs. 7 ± 0.76 months; HR, 0.60; 95% CI, 0.452-0.805; P<0.001). Multivariable analysis identified LT+TKI, vascular invasion, and metastasis as independent risk factors for poorer survival outcomes.

**Conclusions:**

Adding ICI to LT and TKI significantly extends both OS and PFS in patients with unresectable HCC. These findings suggest that integrating ICI into treatment protocols could be beneficial in managing unresectable HCC, particularly for patients with vascular invasion.

## Introduction

Hepatocellular carcinoma (HCC) is the most common primary liver malignancy and represents a significant global health challenge due to its high morbidity and mortality rates, it is the sixth most common cancer worldwide and becomes the fourth leading cause of cancer-related death (1–5). HCC may be treatable in early stages by resection, liver transplantation, or ablation (6). However, patients are typically identified at intermediate or advanced stages due to a lack of symptoms. Despite advances in diagnostic techniques and therapeutic interventions, the prognosis for patients with unresectable HCC remains poor (7, 8).

For the patient with advanced HCC, multiple guidelines recommend sorafenib as the first-line treatment due to its efficacy and safety (9). The evolution of systemic therapies for HCC has notably included the adoption of other tyrosine kinase inhibitors (TKIs) such as lenvatinib, which have become staples in the treatment of unresectable HCC (10).

Recent developments in cancer immunotherapy have introduced immune checkpoint inhibitors (ICIs) that target inhibitory receptors on T cells, such as PD-1 and CTLA-4, thus enhancing the immune system’s ability to eliminate cancer cells (11, 12). Currently, several immune checkpoint inhibitors have been approved by the FDA for the treatment of hepatocellular carcinoma (HCC) (13). In 2022, nivolumab, the first immune checkpoint inhibitor, was approved as a second-line treatment option for patients with sorafenib-pretreated HCC (14). Additionally, pembrolizumab, another PD-1 inhibitor, was approved as an alternative treatment for patients with sorafenib-resistant HCC (15). Prior to the clinical trial of IMbrave150, sorafenib was the standard first-line systemic therapy for unresectable advanced HCC. However, the results of the IMbrave150 trial demonstrated that the combination of atezolizumab and bevacizumab significantly improved OS and PFS compared to sorafenib in patients with unresectable HCC (16). Based on these findings, the Barcelona Clinic Liver Cancer (BCLC) staging system and treatment guidelines now recommend atezolizumab plus bevacizumab as the first-line systemic therapy for advanced stage (C) HCC (7). The integration of ICIs into the treatment landscape of HCC, particularly in combination with TKIs and locoregional therapies such as transarterial chemoembolization (TACE), has opened new avenues for improving clinical outcomes.

However, research on the combination of these three regimens has remained limited. Yuan Y et al. (17) conducted a retrospective study in HCC patients with PVTT who received either triple therapy (TACE + targeted therapy + immune therapy) or TACE alone, the results demonstrate that triple therapy group showed significantly improved OS, PFS and overall response rate (ORR) compare to the TACE group, and the grade 3/4 adverse events rates were similar between the two groups. Jin ZC et al. (18) also conducted a large, multi-center, retrospective study included a total of 1244 patients with advanced HCC who received either TACE+TKI+ICI treatment or only TKI+ICI treatment. The results showed that the PFS and OS for TACE combined with targeted and immune therapy were significantly better than those for the combination of targeted and immune therapy alone. According to the researches above, we believe that for patients with unresectable HCC, the combination of locoregional therapy (LT) and systemic therapy may offer improved disease control and prolonged overall survival. Given the promising outcomes of ICIs when used in combination with TKIs in the clinical trial mentioned above, We hypothesize that the integration of ICI can enhance the antitumor efficacy of traditional dual therapy of LT and TKI. Consequently, we designed this study to evaluate the comparative efficacy of dual therapy, which consists of LT combined with TKI, versus triple therapy of the addition of ICI to the regimen of LT and TKI.

## Materials and methods

### Patient selection

This retrospective analysis involved 232 patients with a diagnosis of unresectable HCC, treated at West China Hospital between January 2019 and December 2023. The diagnosis was established through pre-treatment enhanced computed tomography (CT) or magnetic resonance imaging (MRI), each independently evaluated by two experienced radiologists. All imaging studies were completed within two weeks prior to initiation of treatment. In instances where the imaging-based diagnosis remained ambiguous, ultrasound-guided liver biopsy was employed to ascertain the diagnosis. Criteria for unresectability were determined by a multidisciplinary team and included (1): infeasibility of R0 resection, (2) a remaining liver volume less than 30% in non-cirrhotic patients or less than 40% in cirrhotic patients, and/or an indocyanine green clearance rate exceeding 15%.

Inclusion criteria for the study were as follows: (1) primary unresectable HCC as evidenced by MRI or CT imaging characteristics, (2) Child–Pugh class A or B liver function, and an Eastern Cooperative Oncology Group (ECOG) performance status of 0 or 1, (3) treatment that included both locoregional therapy (LT) and tyrosine kinase inhibitors (TKI), with or without immune checkpoint inhibitors. Exclusion criteria were: (1) presence of any malignant tumor other than HCC, (2) incomplete medical records, (3) loss to follow-up within three months post-treatment.

### Treatment protocols

The patients were divided into two groups. The LT+TKI group underwent standard locoregional treatments such as TACE, stereotactic body radiotherapy (SBRT) or radiofrequency ablation (RFA), followed by first-line tyrosine kinase inhibitors including sorafenib or lenvatinib based on individual clinical evaluations. The LT+TKI+ICI group received identical locoregional and TKI therapies, supplemented with an ICI (sintilimab, tislelizumab, and camrelizumab) administered according to established guidelines.

### Follow-up and monitoring

Patients were monitored at intervals ranging from three to six months following the initial treatment. Clinical evaluations, imaging to evaluate disease progression, tests to monitor liver function and pertinent tumor markers such as AFP or PIVKA-II were conducted regularly. The primary endpoint of this study was overall survival (OS), and the secondary endpoint was progression-free survival (PFS). Both endpoints were calculated from the initiation of treatment to the date of death or the date of first documented disease progression, respectively. Treatment-related adverse events were recorded according to the National Cancer Institute Common Terminology Criteria for Adverse Events 4.0 (19). Patients who were lost to follow-up during the study period were considered censored at their last hospital visit.

### Data analysis

Categorical variables were presented with frequency and percentage. The Pearson chi-square test and Fisher’s exact test were employed for comparing categorical variables between groups. The survival curves of OS and PFS in the entire cohort and subgroup analysis were analyzed utilizing the Kaplan–Meier method with the Log rank test. All statistical tests were two-sided, and P < 0.05 was considered statistically significant. In Cox regression, variables with P <0.1 were included in the multivariate regression, with a P <0.05 considered statistically significant. All data analyses were conducted using R version 4.4.1, and SPSS Version 29.0.1.

## Results

### Baseline characteristics

The study enrolled 232 patients who were evaluated by multidisciplinary team (MDT) experts as having unresectable HCC, comprising 107 individuals who underwent locoregional therapy and tyrosine kinase inhibitor treatment (LT+TKI group) and 125 individuals who underwent locoregional therapy and tyrosine kinase inhibitor plus immune checkpoint inhibitor treatment (LT+TKI+ICI group). The median follow-up time in the LT+TKI group was 16 (range: 9–26) months and 17 (range: 11.5–25) months in the LT+TKI+ICI group. There were 71 (66.4%) death events occurred in the LT+TKI group and 55 (44.0%) in the LT+TKI+ICI group. As presented in **Table 1**, critical baseline data were analyzed, including age, gender, ALT and AST levels, ALBI, Child-Pugh score, vascular invasion, AFP and PIVKA-II levels, the number and size of tumors in the liver, metastasis, CNLC stage, hepatitis and cirrhosis. The baseline characteristics exhibited no significant differences between the two groups, with the exception of gender. Compared to the LT+TKI group, the LT+TKI+ICI group had a lower proportion of female patients enrolled.

**Table 1:** Baseline characteristic of unresectable HCC.

Characteristics	LT+TKI (n=107)	LT+TKI+ICI (n=125)	P value
Age			
≤50	58 (54.2)	72 (57.6)	0.604
>50	49 (45.8)	53 (42.4)	
Gender			
Female	19 (17.8)	11 (8.8)	0.043
Male	88 (82.2)	114 (91.2)	
ALT, U/L			
≤40	60 (56.1)	58 (46.4)	0.142
>40	47 (43.9)	67 (53.6)	
AST, U/L			
≤40	37 (34.6)	46 (36.8)	0.725
>40	70 (65.4)	79 (63.2)	
ALBI			
Grade1	56 (52.3)	72 (57.6)	0.723
Grade2	46 (43.0)	48 (38.4)	
Grade3	5 (4.70)	5 (4.0)	
Child-Pugh score			
A	87 (81.3)	110 (88.0)	0.167
B	18 (16.8)	15 (12.0)	
C	2 (1.9)	0 (0.0)	
Vascular invasion			
No	33 (30.8)	45 (36.0)	0.407
Yes	74 (69.2)	80 (64.0)	
AFP, ng/mL			
≤400	53 (49.5)	63 (50.4)	0.895
>400	54 (50.5)	62 (49.6)	
PIVKA-II, mAU/mL			
≤1000	43 (40.2)	43 (34.4)	0.363
>1000	64 (59.8)	82 (65.6)	
Tumor number			
≤3	28 (26.2)	41 (32.8)	0.271
>3	79 (73.8)	84 (67.2)	
Tumor size			
≤10cm	84 (78.5)	90 (72.0)	0.254
>10cm	23 (21.5)	35 (28.0)	
Intrahepatic metastasis			
No	19 (17.8)	22 (17.6)	0.975
Yes	88 (82.2)	103 (82.4)	
Lymphatic metastasis			
No	83 (77.6)	92 (73.6)	0.484
Yes	24 (22.4)	33 (26.4)	
Metastasis			
No	92 (86.0)	101 (80.8)	0.293
Yes	15 (14.0)	24 (19.2)	
CNLC stage			
Ib	4 (3.7)	7 (5.6)	0.300
IIIa	3 (2.8)	8 (6.4)	
IIb	21 (19.6)	25 (20.0)	
IIIa	61 (57.0)	60 (48.0)	
IIIb	16 (15.0)	25 (20.0)	
IV	2 (1.9)	0 (0.0)	
Cirrhosis			
No	20 (18.7)	21 (16.8)	0.707
Yes	87 (81.3)	104 (83.2)	
Hepatitis			
No	11 (10.3)	13 (10.4)	0.051
HBV	87 (81.3)	110 (88.0)	
HCV	9 (8.4)	2 (1.6)	

ALT, alanine aminotransferase; AST, aspartate aminotransferase; AFP, alpha-fetoprotein; PIVKA-II, protein induced by vitamin K absence II; ALBI, albumin-bilirubin; CNLC, China Liver Cancer Staging; HBV, hepatic B virus; HCV, hepatic C virus.

### OS analysis between the LT+TKI and LT+TKI+ICI groups

**Figure 1:**
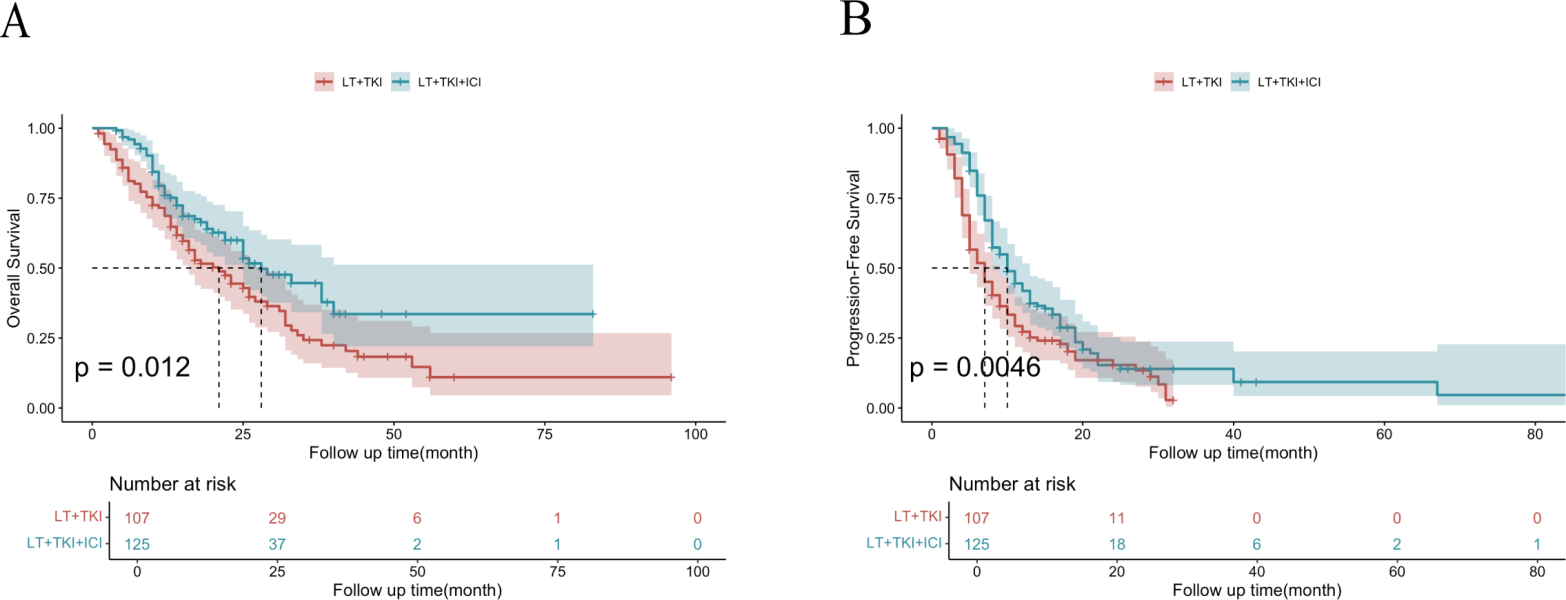
Kaplan-Meier curves of OS **(A)** and PFS **(B)** for the entire cohort of patients in the LT+TKI and LT+TKI+ICI groups.

As shown in **Figure 1A**, the median OS was 21 ± 3.0 and 28 ± 3.9 months in the LT+TKI and LT+TKI+ICI groups, respectively. The 6-, 12-, and 24-month OS rates were 85.8%, 71.5%, and 44.1% in the LT+TKI group, and 96.8%, 79.3%, and 59.4% in the LT+TKI+ICI group, respectively. The LT+TKI+ICI group demonstrated a significantly improved OS compared to the LT+TKI group (HR, 0.64; 95% CI, 0.449-0.913; P = 0.014).

### PFS analysis between the LT+TKI and LT+TKI+ICI groups

As shown in **Figure 1B**, the median PFS was 7 ± 0.76 and 11 ± 1.1 months in the LT+TKI and LT+TKI+ICI groups, respectively. The 3-, 6-, and 12-month PFS rates were 90.6%, 64.0%, and 41.5% in the LT+TKI group, and 97.6%, 86.9%, and 60.5% in the LT+TKI+ICI group, respectively. The LT+TKI+ICI group also demonstrated a significantly longer PFS compared to the LT+TKI group (HR, 0.60; 95% CI, 0.452-0.805; P< 0.001).

### Risk factor of overall survival and progression free survival

Univariable and multivariable analysis of OS are conducted. Multivariable analysis indicate that LT+TKI therapy (HR, 1.72; 95% CI, 1.20-2.46; P = 0.003), vascular invasion (HR, 1.97; 95% CI,1.29-3.02; P = 0.002), lymphatic metastasis (HR, 1.59; 95% CI, 1.08-2.34; P= 0.019), metastasis (HR, 1.74; 95% CI, 1.14-2.66; P = 0.01), and PIVKA >1000mAU/mL (HR, 1.50; 95% CI,1.01-2.24; P = 0.045) were independent risk factors associated with poorer OS (**Table 2**).

**Table 2:** Univariable and multivariable analysis of variable for OS.

variable	univariable analysis		multivariable analysis	
	HR(95%CI)	P value	HR(95%CI)	P value
Age				
>50 vs ≤50	0.59 (0.41-0.85)	0.004	0.73 (0.49-1.08)	0.117
Gender				
Male vs Female	0.95 (0.58-1.55)	0.83		
ALT				
>40 vs ≤40	1.29 (0.91-1.86)	0.151		
AST				
>40 vs ≤40	1.47 (1.01-2.15)	0.042	1.19 (0.81-1.74)	0.386
ALBI				
Grade2 vs Grade1	1.33 (0.93-1.91)	0.116		
Grade3 vs Grade1	2.06 (0.83-5.16)	0.121		
Child-Pugh score				
B vs A	1.27 (0.78-2.04)	0.334		
C vs A	1.86 (0.26-13.38)	0.539		
AFP				
>400 vs ≤400	1.58 (1.11-2.26)	0.011	1.27 (0.87-1.85)	0.223
PIVKA-II				
>1000 vs ≤1000	1.71 (1.17-2.50)	0.006	1.50 (1.01-2.24)	0.045
Tumor number				
>3 vs ≤3	1.57 (1.03-2.41)	0.037	1.15 (0.74-1.79)	0.526
Tumor size				
>10cm vs ≤10cm	1.21 (0.80-1.82)	0.372		
Vascular invasion				
Yes vs No	2.26 (1.50-3.41)	<0.001	1.97 (1.29-3.02)	0.002
Intrahepatic metastasis				
Yes vs No	1.09 (0.67-1.78)	0.733		
Lymphatic metastasis				
Yes vs No	1.85 (1.27-2.70)	0.001	1.59 (1.08-2.34)	0.019
Metastasis				
Yes vs No	1.96 (1.30-2.96)	0.001	1.74 (1.14-2.66)	0.01
Cirrhosis				
Yes vs No	0.81 (0.51-1.3)	0.379		
Hepatitis				
HBV vs No	0.69 (0.41-1.17)	0.172		
HCV vs No	0.74 (0.30-1.79)	0.498		
Treatment				
LT+TKI vs LT+TKI+ICI	1.56 (1.10-2.23)	0.014	1.72 (1.20-2.46)	0.003

ALT, alanine aminotransferase; AST, aspartate aminotransferase; AFP, alpha-fetoprotein; PIVKA-II, protein induced by vitamin K absence II; ALBI, albumin-bilirubin; HBV, hepatic B virus; HCV, hepatic C virus.

Univariable and multivariable analysis of PFS are conducted as well. Multivariable analysis indicate that LT+TKI therapy (HR, 1.65; 95% CI, 1.24-2.21; P< 0.001), metastasis (HR, 1.50; 95% CI, 1.04-2.18; P = 0.032), age < 50 (HR, 1.38; 95% CI,1.01-1.88; P = 0.041) were independent risk factors associated with poorer PFS (**Supplementary Table S1**).

### Subgroup analysis on the prognosis of macrovascular invasion

**Figure 2:**
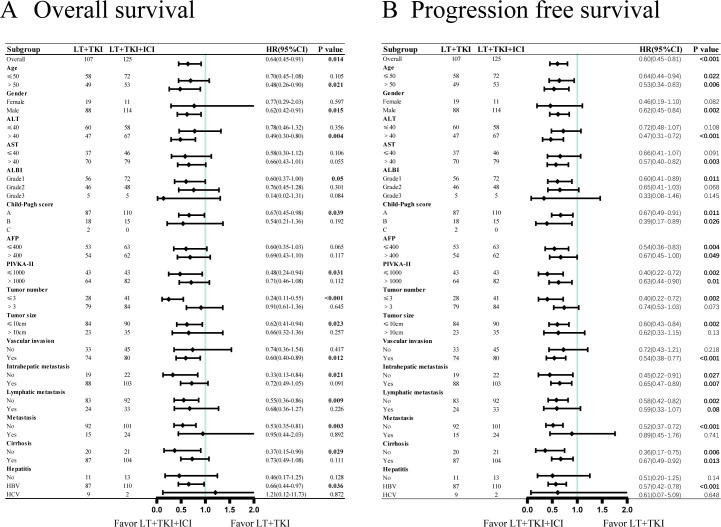
Subgroup analyses of **(A)** overall survival and **(B)** progression-free survival in the patient subgroups.

Forest plot analysis in subgroups was conducted. The results indicate a significantly improved OS in the LT+TKI+ICI group compared to the LT+TKI group for patients with the following characteristics: age >50, male, ALT >40u/ml, ALBI grade 1, Child-Pugh score class A, PIVKA-II ≤ 1000 mAu/ml, number of tumors ≤3, size of tumor ≤10cm, vascular invasion, no intrahepatic metastasis, no lymphatic metastasis, no extrahepatic metastasis, no cirrhosis, and HBV infection (**Figure 2A**). PFS was significantly prolonged in the LT+TKI+ICI group compared to the LT+TKI group for patients who were male, had ALT >40u/ml, AST >40u/ml, ALBI grade 1, number of tumors ≤3, size of tumor ≤10cm, vascular invasion, no extrahepatic metastasis, and HBV infection (**Figure 2B**).

**Figure 3:**
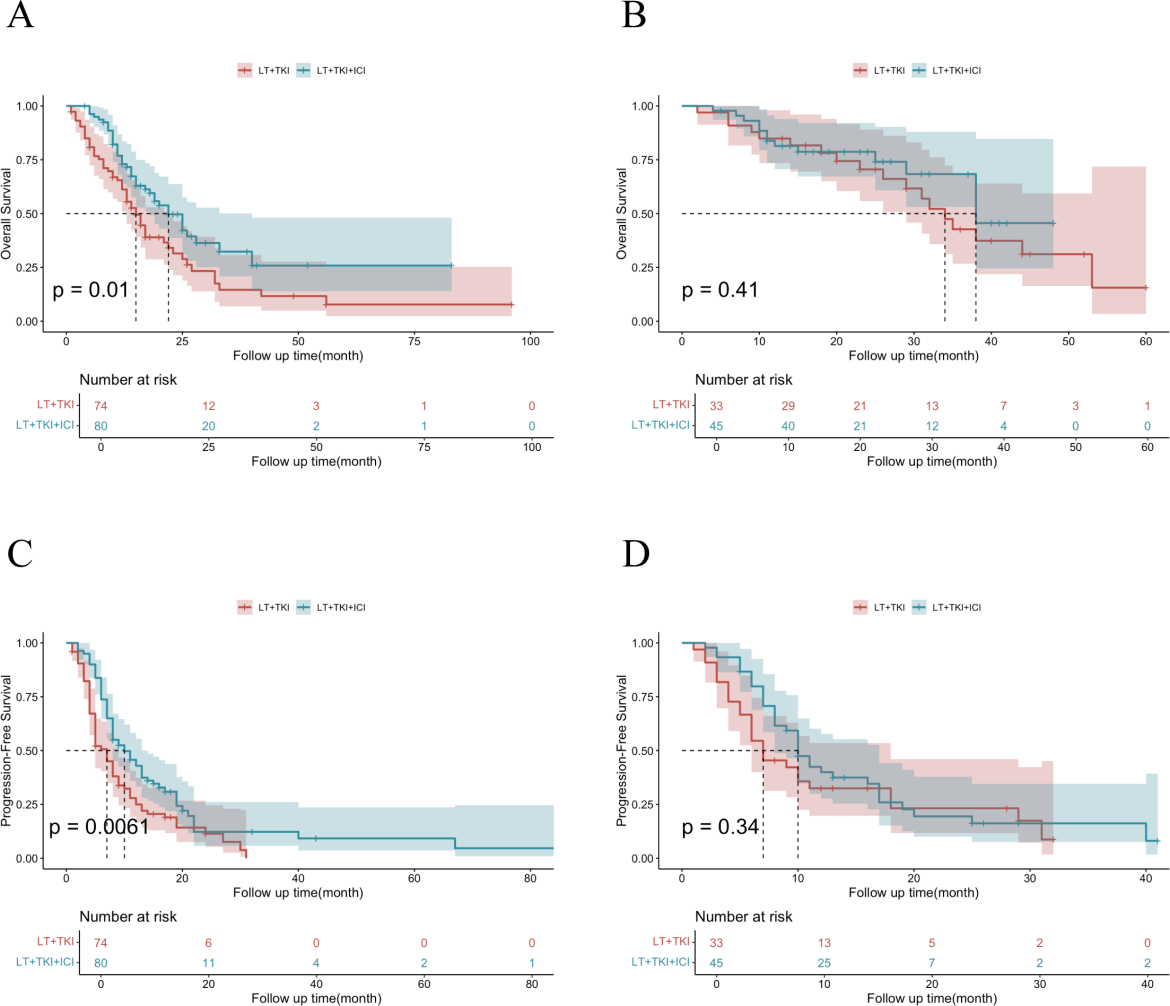
Survival analysis of subgroups stratified by Vascular Invasion.Kaplan-Meier curves of OS in patients with vascular invasion **(A)** and without vascular invasion **(B)** in the LT+TKI and LT+TKI+ICI groups. Kaplan-Meier curves of PFS in patients with vascular invasion **(C)** and without vascular invasion **(D)** in the LT+TKI and LT+TKI+ICI groups.

To further elucidate the impact of vascular invasion on prognosis, we conducted a subgroup analysis comprising 78 individuals without vascular invasion and 154 individuals with vascular invasion. The median OS of patients with vascular invasion was 15.0 ± 1.63 and 22.0 ± 2.78 months in the LT+TKI group and LT+TKI+ICI group, respectively, and 34.0 ± 2.86 and 38.0 ± 2.71 months in the two groups, respectively, without vascular invasion. The results indicated that the addition of ICI led to a significantly improved OS in patients with vascular invasion (HR, 0.60; 95% CI, 0.40-0.89; P=0.012) (**Figure 3A**). However, in the subgroup of patients without vascular invasion, the OS of the LT+TKI group and LT+TKI+ICI group showed no significant difference (HR, 0.74; 95% CI, 0.36-1.54; P=0.417) (**Figure 3B**). The PFS analysis demonstrated a similar trend, with the median PFS of patients with vascular invasion being 6.0 ± 0.65 and 10.0 ± 1.02 months in the LT+TKI group and LT+TKI+ICI group, respectively, and 11.0 ± 2.06 and 12.0 ± 1.39 months in the two groups, respectively, without vascular invasion. The addition of ICI also prolonged PFS in patients with vascular invasion (HR, 0.54; 95% CI, 0.38-0.77; P<0.001) (**Figure 3C**). In the subgroup of patients without vascular invasion, the PFS of the LT+TKI group and LT+TKI+ICI group showed no significant difference (HR, 0.72; 95% CI, 0.43-1.21; P=0.218) (**Figure 3D**).

We also conducted a subgroup analysis stratified by PIVKA-II level and extrahepatic metastasis. In the subgroup of different PIVKA-II level, the results indicated that the addition of ICI led to a significantly improved OS in patients with low PIVKA-II level (40 vs 26 months; P=0.026) (**Supplementary Figure S1A**). However, in the subgroup of patients with high PIVKA-II level, the OS of the LT+TKI group and LT+TKI+ICI group showed no significant difference (25 vs 17 months; P=0.10) (**Supplementary Figure S1B**). Nevertheless, the PFS analysis demonstrated a same trend, the addition of ICI also prolonged PFS in patients with low PIVKA-II level (11 vs 6 months; P=0.0098) (**Supplementary Figure S1C**). In the subgroup of patients with high PIVKA-II level, the PFS of the LT+TKI group and LT+TKI+ICI group showed no significant difference (10 vs 7 months; P=0.11) (**Supplementary Figure S1D**). And in the subgroup of extrahepatic metastasis, the results indicated that the addition of ICI led to a significantly improved OS in patients without extrahepatic metastasis (40 vs 23 months; P=0.0022) (**Supplementary Figure S2B**). However, in the subgroup of patients who have extrahepatic metastasis, the OS of the LT+TKI group and LT+TKI+ICI group showed no significant difference (12 vs 12 months; P=0.89) (**Supplementary Figure S2A**). However, the PFS analysis demonstrated a contrast trend, the addition of ICI led to a significantly improved PFS in patients with extrahepatic metastasis (8.5 vs 4 months; P<0.001) (**Supplementary Figure S2C**). However, in the subgroup of patients without extrahepatic metastasis, the PFS of the LT+TKI group and LT+TKI+ICI group showed no significant difference (11 vs 8 months; P=0.051) (**Supplementary Figure S2D**).

### Treatment-related adverse events

The adverse events in the two groups are listed in **Table 3**. Overall, no death events occurred because of Treatment-related adverse events (TRAEs) during the follow-up period and there was no significant difference between the LT+TKI and LT+TKI+ICI groups in the types of severe TRAEs. The incidence of severe treatment-related adverse events (TRAEs) remained low in both groups, and patients who reported severe TRAEs temporarily discontinued TKI or ICI administration until the adverse effects were alleviated or resolved.

**Table 3:** Treatment-related adverse event in the entire cohort.

Adverse events	Any grades			Grades 3/4		
	LT+TKI (n=107)	LT+TKI+ICI (n=125)	P value	LT+TKI (n=107)	LT+TKI+ICI (n=125)	P value
Hypertension	17 (15.9)	18 (14.4)	0.752	3 (2.8)	4 (3.2)	0.86
Hand–foot skin reaction	29 (27.1)	33 (26.4)	0.904	5 (4.7)	7 (5.6)	0.751
Bone marrow suppression	40 (37.4)	50 (40.0)	0.683	7 (6.5)	9 (7.2)	0.844
Vomiting	72 (67.3)	82 (65.6)	0.786	3 (2.8)	2 (1.6)	0.529
Rash	15 (14.0)	23 (18.4)	0.369	0	0	/
Diarrhea	36 (33.6)	43 (34.4)	0.904	5 (4.7)	5 (4.0)	0.801
Hypothyroidism	14 (13.1)	24 (19.2)	0.210	1 (0.9)	5 (4.0)	0.143
Elevated ALT	48 (44.9)	57 (45.6)	0.910	11 (10.3)	12 (9.6)	0.863
Elevated AST	56 (52.3)	64 (51.2)	0.863	14 (13.1)	18 (14.4)	0.772
Hyperbilirubinemia	61 (57.0)	73 (58.4)	0.831	5 (4.7)	7 (5.6)	0.751
Elevated creatinine	15 (14.0)	27 (21.6)	0.135	4 (3.7)	6 (4.8)	0.691
Abdominal pain	29 (27.1)	28 (22.4)	0.407	0	0	/

ALT, alanine aminotransferase; AST, aspartate aminotransferase.

## Discussion

The present study rigorously evaluated the efficacy of adding an ICI to the established regimen of locoregional therapy and TKI in patients with unresectable HCC. Our findings demonstrate a significant improvement in both OS and PFS in the group receiving the combined therapy of LT+TKI+ICI, compared to those who received only LT+TKI therapy. Specifically, the median OS increased from 21 months in the LT+TKI group to 28 months in the LT+TKI+ICI group, accompanied by a superior PFS of 11 months compared to 7 months in the LT+TKI group. Further subgroup analyses revealed that patients with vascular invasion in the unresectable HCC cohort might derive greater benefits from the addition of ICI. These results indicate the potential of ICIs to enhance the therapeutic landscape for HCC, suggesting that combining immunotherapy with locoregional therapy and TKI may provide a more effective strategy for the treatment of unresectable HCC.

For the treatment of unresectable HCC, the only proven therapy was sorafenib, a tyrosine kinase inhibitor. Recently, the synergistic effect of an immune checkpoint inhibitor, atezolizumab plus bevacizumab outperformed sorafenib alone in terms of survival, making it the recommended first-line therapy. Other multikinase inhibitors, lenvatinib and regorafenib, were also recommended as first and second-line drugs, respectively (6, 20–22). Ann-Lii Cheng et al. (16) reported that a combination of atezolizumab and bevacizumab significantly improved both OS(19.2 months [95% CI 17.0–23.7] vs 13.4 months [95% CI 11.4–16.9]) and PFS (6.9 months [95% CI 5.7-8.6] vs 4.3 months [95% CI 4.0-5.6]) compared to the monotherapy with sorafenib. These findings highlight the potential of immunotherapy as a pivotal component of future therapeutic strategies for advanced HCC. Shen et al. (23) reported enhanced disease control and survival rates when combining TACE with sintilimab and lenvatinib, supporting the notion that multi-modal therapy can be beneficial. Similarly, Wang et al. (24) observed improved outcomes in infiltrative HCC with the addition of ICIs to hepatic arterial infusion chemotherapy and lenvatinib.

These studies above indicate the multiple therapy may enhanced the efficacy of individual treatments. HCC and other solid tumor cells can avoid the immune system attack by inducing the expression of immune checkpoints in T cells. ICIs are monoclonal antibodies that could block the interaction of immune checkpoint proteins with their ligands, thereby enhance the anti-tumor immune response by preventing the inactivation of T cells and restoring immune recognition and immune attack (25, 26). Historically, it was presumed that immune interventions were incompatible with conventional chemotherapies. However, emerging evidence indicates that standard chemotherapy agents might actually induce immunogenicity in the tumor microenvironment and the immune system itself. For instance, chemotherapeutic agents such as oxaliplatin, frequently utilized in TACE or hepatic artery infusion chemotherapy (HAIC), have been shown to upregulate HLA class I expression in tumor cells, associated with the proliferation and activation of cytotoxic T cells (27). Furthermore, TACE could induce tissue hypoxia that results in the upregulation of vascular endothelial growth factor (VEGF), which may lead to tumor revascularization and local recurrence (28), TKIs target the VEGF pathway, downregulate the VEGF expression, potentially inducing vascular normalization. Consequently, the combination of TACE with anti-angiogenic agents may potentially delay tumor progression or recurrence (17). In addition, the vascular normalization can enhance blood perfusion and reduce vascular leakage, leading to improved CD8+ T-cell infiltration in tumor tissues, as demonstrated in various animal models (29–32), which may enhance the efficacy of immune therapy theoretically. Additionally, ICIs can enhance anti-tumor activity by activating CD8+ T-cell function, thereby improving the tumor-killing efficacy of LT and TKIs. Based on these insights, the combination of a triple therapy regimen combining ICIs, LT, and TKIs may be crucial for maximizing therapeutic outcomes in advanced unresectable HCC.

Numerous studies have focused on the integration of ICIs with traditional therapies for HCC to investigate potential improvements in survival outcomes for patients ineligible for curative resection. Kelley RK et al. (33) enrolled the 837 patients who were randomly assigned to combination treatment of cabozantinib plus atezolizumab (n=432), sorafenib (n=217), or single-agent cabozantinib (n=188), the median progression-free survival was 6.8 months (99% CI 5.6-8.3) in the combination treatment group versus 4.2 months (2.8-7.0) in the sorafenib group (HR 0.63, 99% CI 0.44-0.91, P=0.0012). Median overall survival was 15.4 months (96% CI 13.7-17.7) in the combination treatment group versus 15.5 months (12.1-not estimable) in the sorafenib group (HR 0.90, 95% CI 0.69-1.18; P=0.44). Locoregional therapy such as TACE or ablation were considered suitable for patients with intermediate stage HCC. However, it has also been utilized extensively beyond this stage and has become a commonly employed non-surgical treatment option for various stages of HCC due to its effectiveness and widespread availability (23). The previous study shows TACE plus Lenvatinib and sintilimab leads to a satisfied median overall survival of 23.6 months (95%CI 22.2-25.0 months) (34). Jin ZC et al. (18) analysis 1244 patients received ICI-VEGF with or without TACE. the result showed TACE-ICI-VEGF group exhibited a significantly improved median OS (22.6 months [95% CI: 21.2-23.9] vs 15.9 months [14.9-17.8]; P < 0.0001). In our study, median OS was 28 ± 3.9 months in the LT+TKI+ICI group versus 21 ± 3.0 months in the LT+TKI group (HR, 0.64; 95% CI, 0.449-0.913; P = 0.014). Median PFS also favored the LT+TKI+ICI group (11 ± 1.1 months vs 7 ± 0.76 months; HR, 0.60; 95% CI, 0.452-0.805; P<0.001). Based on the findings, the combination of LT, TKI and ICI shows benefits in the treatment of unresectable HCC.

In our study cohort, patients with vascular invasion appeared to derive greater benefit from treatment compared to those without such invasion. Shen L et al. (35)reported TACE plus SBRT could provide improved OS and PFS in the patients with macrovascular invasion. SBRT is an advanced radiation modality that can concentrate a high radiation dose precisely on the tumor in a few fractions, provide a high local control rate (>80%) for tumor thrombosis, while TACE provides good control of tumors outside the radiation field as a complement (36). Additionally, the incorporation of a TKI and an ICI may further enhance control over the systemic tumor burden and improve the efficacy of locoregional therapies (8).

While our study provides valuable insights into the benefits of combining ICIs with locoregional therapies and TKIs in treating unresectable HCC, it has several limitations. As a retrospective analysis, our study is subject to inherent biases associated with such study designs, including selection and information bias, because the treatment was determined by doctors’ clinical judgement, patients’ tolerance and family economic affordability. We tried our best to minimize this bias by enrolling patients with specific inclusion and exclusion criteria, and all enrolled patients have been evaluated by a fixed-member MDT team to ensure treatment consistency. However, prospective randomized controlled trials are still needed to validate our findings. Our data derived from a single institution may not be generalizable to a broader population due to specific demographic and treatment practice variations. The relatively short follow-up period may not fully capture long-term survival outcomes and late emerging effects of the therapy combinations.

## Conclusion

Integrating immune checkpoint inhibitors (ICIs) with locoregional therapies (LT) and tyrosine kinase inhibitors (TKIs) signifies a major advancement in treating unresectable hepatocellular carcinoma (HCC). This study highlights a triple therapy regimen’s potential to improve survival outcomes for advanced unresectable HCC patients, emphasizing ICIs’ role in enhancing established treatments. Our findings may set the stage for future investigations into the integration of immunotherapy with traditional HCC therapies, potentially leading to more refined and effective treatment protocols for this challenging condition.

## Data Availability

The raw data supporting the conclusions of this article will be made available by the authors, without undue reservation.
