# Supplementary figures and images for: Enhanced efficacy of immune checkpoint inhibitors combined locoregional therapy and tyrosine kinase inhibitors in the treatment of unresectable hepatocellular carcinoma: A single - center retrospective study
Source: Front Oncol. 2025 Feb 25;15:1554711. doi: 10.3389/fonc.2025.1554711 (PMC11893395; doi:10.3389/fonc.2025.1554711)

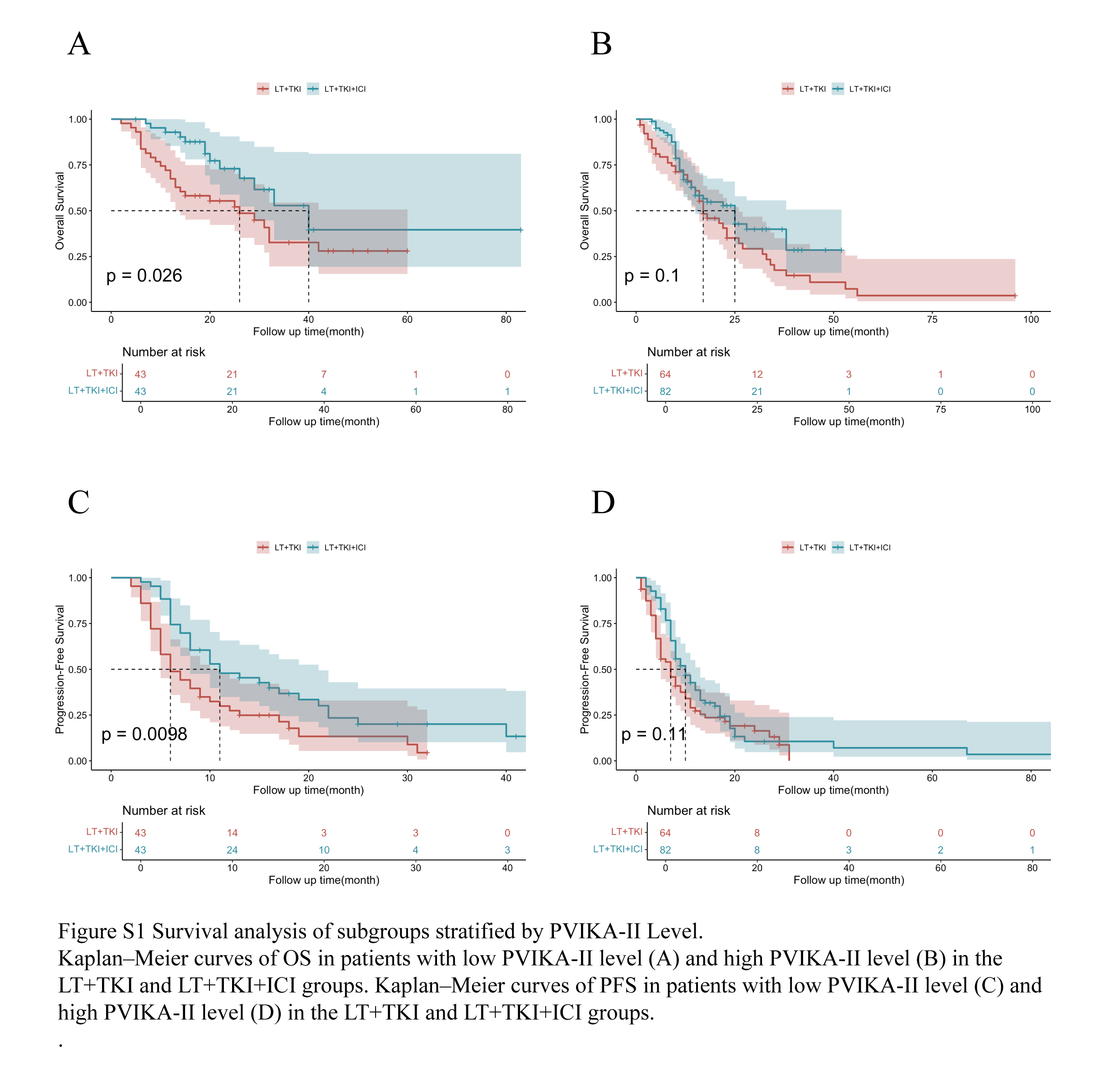

Supplement: Supplementary file 1 [file Image1.tif]

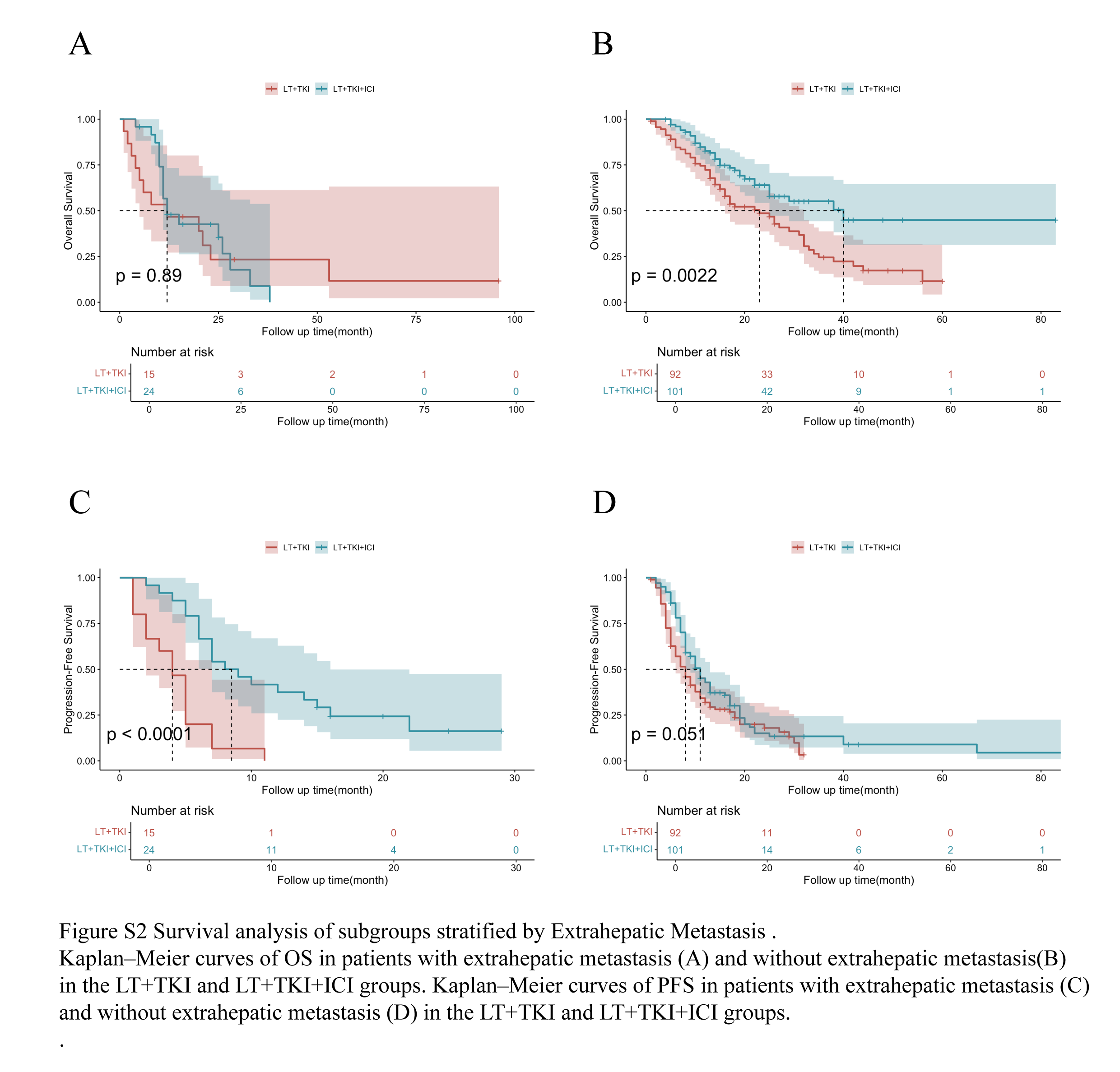

Supplement: Supplementary file 2 [file Image2.tif]
