# Supplementary material for: Enhanced efficacy of immune checkpoint inhibitors combined locoregional therapy and tyrosine kinase inhibitors in the treatment of unresectable hepatocellular carcinoma: A single - center retrospective study
Source: Front Oncol. 2025 Feb 25;15:1554711. doi: 10.3389/fonc.2025.1554711 (PMC11893395; doi:10.3389/fonc.2025.1554711)
Supplement: Supplementary file 3 [file Table1.docx]

TableS1 Univariable and Multivariable Analysis of variable for PFS

| variable | univariable analysis | | multivariable analysis | |
| --- | --- | --- | --- | --- |
|  | HR(95%CI) | P value | HR(95%CI) | P value |
| **Age** |  |  |  |  |
| ＞50 vs ≤50 | 0.68(0.50-0.89) | **0.007** | 0.73(0.53-0.99) | **0.041** |
| **Gender** |  |  |  |  |
| Male vs Female | 0.80(0.53-1.21) | 0.295 |  |  |
| **ALT** |  |  |  |  |
| ＞40 vs ≤40 | 0.98(0.74-1.31) | 0.914 |  |  |
| **AST** |  |  |  |  |
| ＞40 vs ≤40 | 1.18(0.88-1.59) | 0.273 |  |  |
| **ALBI** |  |  |  |  |
| Grade2 vs Grade1 | 1.15(0.86-1.55) | 0.349 |  |  |
| Grade3 vs Grade1 | 1.44(0.70-2.97) | 0.32 |  |  |
| **Child-Pugh score** |  |  |  |  |
| B vs A | 1.14(0.74-1.71) | 0.576 |  |  |
| C vs A | 1.96(0.48-7.91) | 0.347 |  |  |
| **AFP** |  |  |  |  |
| ＞400 vs ≤400 | 1.47(1.10-1.96) | **0.01** | 1.32(0.98-1.78) | 0.069 |
| **PIVKA-II** |  |  |  |  |
| ＞1000 vs ≤1000 | 1.21(0.90-1.64) | 0.204 |  |  |
| **Tumor number** |  |  |  |  |
| ＞3 vs ≤3 | 1.48(1.07-2.06) | **0.019** | 1.26(0.90-1.76) | 0.179 |
| **Tumor size** |  |  |  |  |
| ＞10cm vs ≤10cm | 0.95(0.67-1.33) | 0.75 |  |  |
| **Vascular invasion** |  |  |  |  |
| Yes vs No | 1.45(1.06-1.97) | **0.018** | 1.30(0.94-1.78) | 0.11 |
| **Intrahepatic metastasis** | |  |  |  |
| Yes vs No | 0.97(0.67-1.41) | 0.873 |  |  |
| **Lymphatic metastasis** |  |  |  |  |
| Yes vs No | 1.32(0.95-1.83) | 0.105 |  |  |
| **Metastasis** |  |  |  |  |
| Yes vs No | 1.64(1.14-2.37) | **0.008** | 1.50(1.04-2.17) | **0.032** |
| **Cirrhosis** |  |  |  |  |
| Yes vs No | 0.89(0.60-1.30) | 0.537 |  |  |
| **Hepatitis** |  |  |  |  |
| HBV vs No | 0.96(0.61-1.56) | 0.916 |  |  |
| HCV vs No | 0.79(0.35-1.81) | 0.58 |  |  |
| **Treatment** |  |  |  |  |
| LT+TKI vs LT+TKI+ICI | 1.66(1.24-2.22) | **<0.001** | 1.65(1.24-2.21) | **<0.001** |

Abbreviations: ALT, alanine aminotransferase; AST, aspartate aminotransferase; AFP, alpha-fetoprotein; PIVKA-II, protein induced by vitamin K absence II; ALBI, albumin-bilirubin; HBV, hepatic B virus; HCV, hepatic C virus.
